# Esophageal Contrast Transesophageal Echocardiography for Mitral Valve Transcatheter Edge-to-Edge Repair in Severe Esophageal Achalasia

**DOI:** 10.1016/j.jaccas.2025.103520

**Published:** 2025-06-04

**Authors:** Keita Shibata, Naoko Ikeda, Tomoyuki Ishinaga, Kohei Wakabayashi, Kaoru Tanno

**Affiliations:** Division of Cardiology, Cardiovascular Center, Showa University Koto-Toyosu Hospital, Tokyo, Japan

**Keywords:** echocardiography, imaging, mitral valve

## Abstract

**Image:**

Severe esophageal achalasia complicates transesophageal echocardiography (TEE), particularly in patients requiring mitral valve transcatheter edge-to-edge repair. We developed an innovative imaging technique called esophageal contrast TEE (ECTE), specifically designed for optimal imaging in complex conditions.

**Case Summary:**

An 80-year-old man with chronic atrial fibrillation and severe mitral regurgitation was referred for mitral transcatheter valve edge-to-edge repair. His severe achalasia with esophageal dilation was challenging for adequate TEE because of poor probe-mucosa contact. Using ECTE, we optimized the probe position and enabled high-quality procedural guidance.

**Take-Home Message:**

ECTE can overcome imaging barriers in severe esophageal pathologies and enable safe and effective cardiac interventions.

## Graphical Abstract

**Figure:**
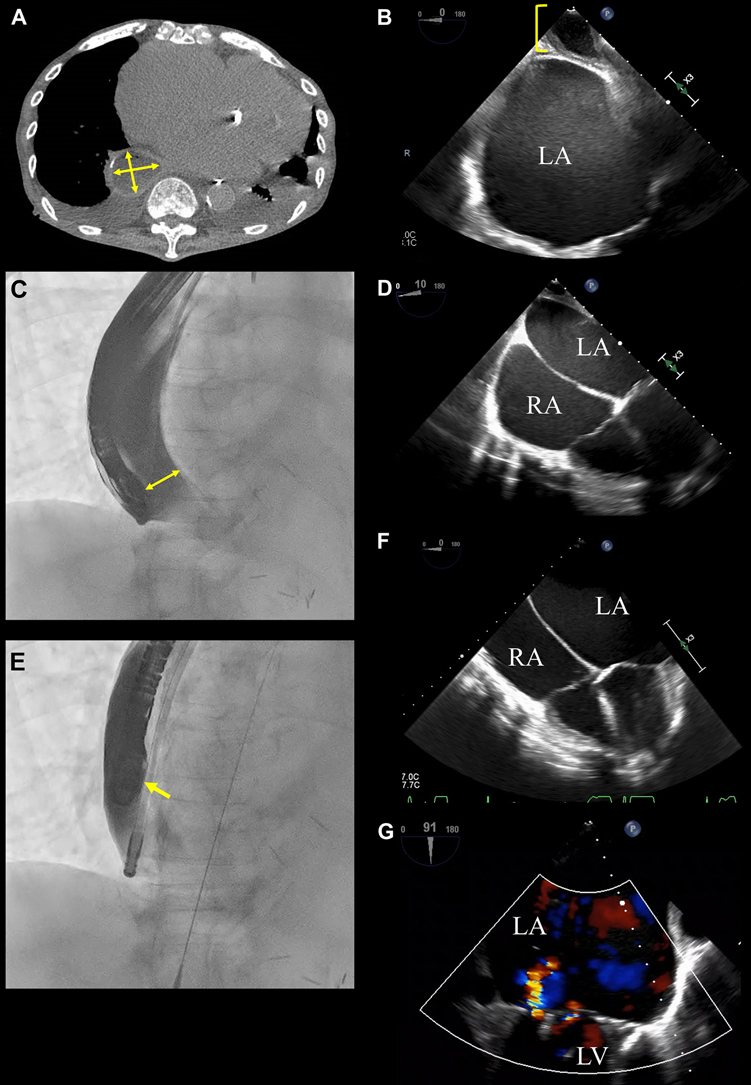

An 80-year-old man was admitted to our hospital with heart failure secondary to severe mitral regurgitation caused by chronic atrial fibrillation. Following a heart team conference, we decided to perform mitral valve transcatheter edge-to-edge repair to address the patient’s heart failure. His medical history included severe achalasia (Eckardt score of 8), which was previously managed with routine balloon dilation for esophageal obstruction, even after peroral endoscopic myotomy. Preoperative computed tomography revealed significant esophageal dilation (4 cm), raising concerns about optimal guidance with transesophageal echocardiography (TEE) during the procedure (Figure 1A).

**Take-Home Message**

• ECTE can overcome imaging barriers in severe esophageal pathologies and enable safe and effective cardiac interventions.

**Figure 1:**
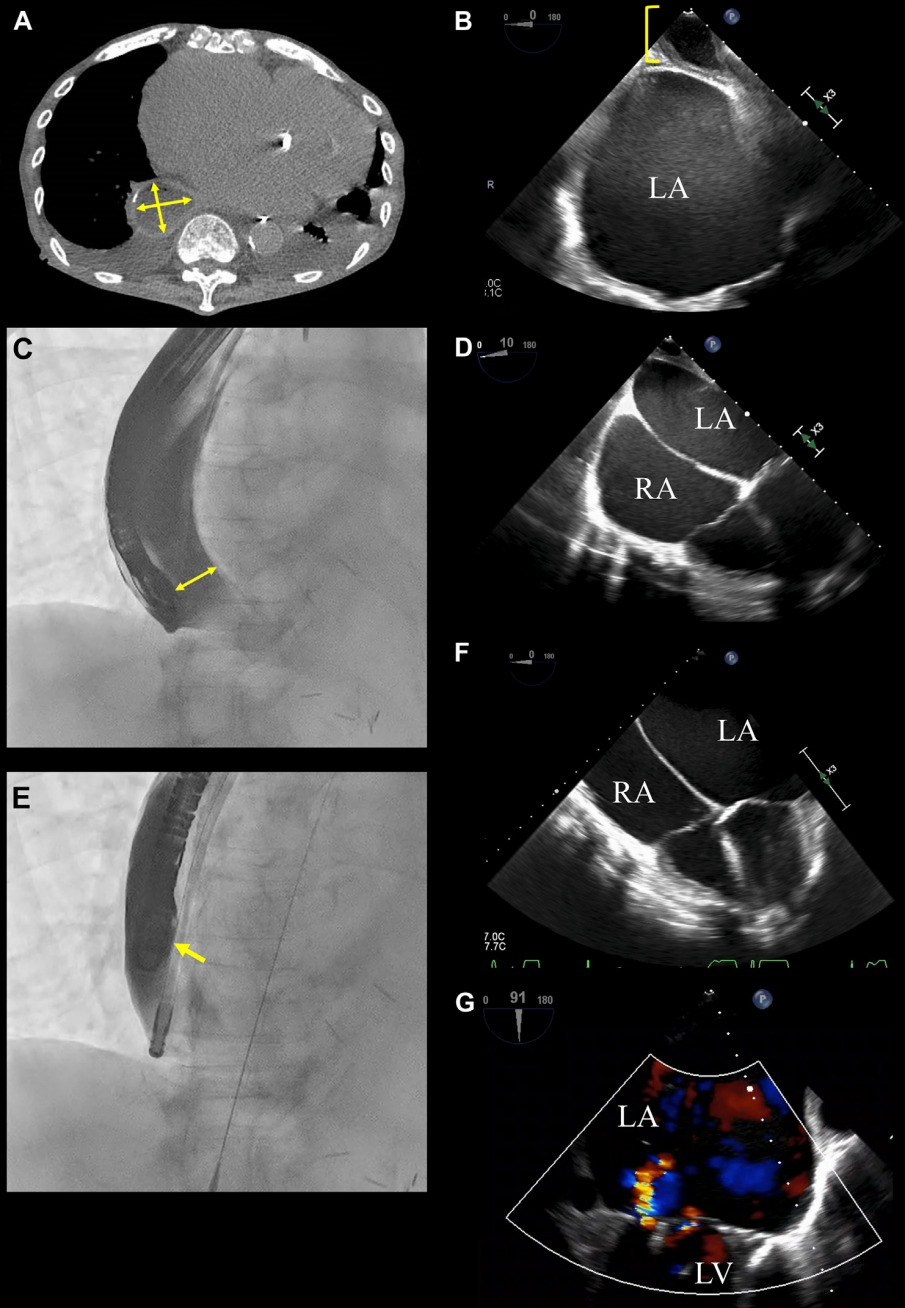
Visualization and Management of Severe Esophageal Achalasia During Transesophageal Echocardiography for Mitral Valve Transcatheter Edge-to-Edge Repair (A) Computed tomography shows significant dilation of the midesophagus approximately 4 × 3.5 cm in diameter (yellow arrows). (B) Transesophageal echocardiography during probe insertion demonstrating approximately 2.5 cm of residual esophageal contents located between the heart and esophageal wall, despite adherence to fasting protocols. (C) Fluoroscopic image obtained during esophageal contrast imaging. The image reveals substantial dilation of the midesophagus measuring approximately 4 cm, with a 2.5-cm gap between the probe and esophageal mucosa (yellow arrow). (D) Predecompression transesophageal echocardiographic image in the 4-chamber view shows the presence of residual esophageal contents, reducing the clarity of mitral valve visualization. (E) Fluoroscopic image after decompression using a gastric tube. The distance between the probe and esophageal mucosa has been reduced to nearly zero, indicating successful decompression (yellow arrow). (F) Postdecompression transesophageal echocardiographic image in the 4-chamber view shows the absence of residual esophageal contents, leading to clearer and improved visualization of the mitral valve. (G) Transesophageal echocardiographic image obtained after mitral valve transcatheter edge-to-edge repair, showing a significant reduction to mild-to-moderate mitral regurgitation. LA = left atrium; LV = left ventricle; RA = right atrium.

We addressed this challenge by combining esophageal contrast imaging with TEE, following advice from gastroenterologists. This strategy allowed continuous monitoring of the spatial relationship between the esophagus and TEE probe during the procedure, ensuring optimal positioning and imaging quality.

After inserting a 12-F gastric tube alongside the TEE probe, residual esophageal contents, likely food remnants despite fasting (Figure 1B), were evacuated before the contrast study. Contrast imaging with 200 mL Gastrografin (Bracco) revealed persistent esophageal dilation and a 2.5-cm gap between the probe and esophageal mucosa (Figures 1C and 1D). Aspiration of the contrast medium through the gastric tube reduced the esophageal diameter, enabling firm contact between the probe and esophageal wall (Figure 1E). This adjustment virtually eliminated the probe-to-heart distance and enhanced the echocardiographic imaging quality by reducing ultrasound attenuation caused by the excess distance (Figure 1F).

With the enhanced imaging provided by the combined TEE and esophageal contrast approach, we successfully performed percutaneous mitral valve repair without complications. Postprocedural TEE confirmed a significant reduction in the severity of mitral regurgitation from severe to mild to moderate (Figure 1G). The patient was discharged and remained free from heart failure–related hospitalizations. Unfortunately, he died of COVID-19 pneumonia 1 year later.

To our knowledge, this is the first reported case of severe achalasia treated for structural heart disease. This case underscores the challenges of performing TEE in patients with significant esophageal pathologies such as severe achalasia. In this patient, pronounced esophageal dilation posed both diagnostic and procedural challenges, with insufficient contact between the TEE probe and esophageal wall, compromising imaging quality and increasing the risk for procedural failure. Accordingly, we developed an innovative imaging technique called esophageal contrast TEE (ECTE), specifically designed for optimal imaging in complex cases like this patient.

Severe achalasia, characterized by esophageal dilation and motility dysfunction, not only affects gastrointestinal health and causes left atrial compression but also impairs cardiac imaging.^1^, ^2^, ^3^ This case demonstrates that ECTE can effectively overcome these challenges by ensuring adequate probe contact and significantly improving imaging precision. Potential complications such as aspiration pneumonia and esophageal perforation must be considered, and thus gastroenterologist consultation is recommended for off-label use. This technique might also aid in cases of hiatal hernia, esophageal motility disorders, and other challenges to ensure safety and optimal imaging.

In conclusion, ECTE offers an effective solution to anatomical challenges and enables safe and precise cardiac intervention. As the complexity of cardiovascular procedures continues to increase, technical innovations are essential for achieving optimal outcomes.

## Funding Support and Author Disclosures

The authors have reported that they have no relationships relevant to the contents of this paper to disclose.
